# Draft Genome Sequence of Pseudomonas sp. nov. H2

**DOI:** 10.1128/genomeA.00241-15

**Published:** 2015-04-02

**Authors:** Wesley Loftie-Eaton, Haruo Suzuki, Kelsie Bashford, Holger Heuer, Pieter Stragier, Paul De Vos, Matthew L. Settles, Eva M. Top

**Affiliations:** a Department of Biological Sciences, University of Idaho, Moscow, Idaho, USA; b Institute for Bioinformatics and Evolutionary Studies (IBEST), University of Idaho, Moscow, Idaho, USA; c Graduate School of Science and Engineering, Yamaguchi University, Yamaguchi, Japan; d Laboratory of Microbiology, Ghent University, Ghent, Belgium

## Abstract

We report the draft genome sequence of Pseudomonas sp. nov. H2, isolated from creek sediment in Moscow, ID, USA. The strain is most closely related to Pseudomonas putida. However, it has a slightly smaller genome that appears to have been impacted by horizontal gene transfer and poorly maintains IncP-1 plasmids.

## GENOME ANNOUNCEMENT

Pseudomonas sp. nov. H2 is a member of the Gammaproteobacteria. The strain was isolated from creek sediment in Moscow, ID, as a transconjugant after a plate mating of a sediment sample with an auxotrophic donor of the IncP-1β plasmid pB10. Transconjugants were selected on M9 medium supplemented with 0.1% gluconic acid and the antibiotics tetracycline (20 mg/liter) and amoxicillin (150 mg/liter) (1). Based on its 16S rRNA sequence, it was provisionally called P. putida H2. While P. putida strains are generally quite capable of maintaining IncP-1 plasmids, strain H2 was reported as an unfavorable host to broad-host-range IncP-1 plasmids, and as such has been used in several plasmid-host evolution and persistence studies (1–4).

Genomic DNA (gDNA) was isolated using a GenElute bacterial genomic DNA kit (Sigma-Aldrich) as per manufacturer’s instructions. The gDNA was sequenced using a whole-genome shotgun approach with paired 150 bp reads generated on MiSeq (Illumina) at the IBEST Genomics Resources Core at the University of Idaho, ID, USA. Sequencing adapters and low-quality bases were trimmed using custom scripts, and reads were assembled using Newbler v2.6. A total of 95 contigs >500 bp were produced (total number of contigs is 132). Of these, the largest was 281,547 bp and the *N*_50_ contig size was 127,674 bp.

Strain H2 was identified as a potential new Pseudomonas species using an in-house, four-gene (*glnA, gyrB, rpoB*, and *rpoD*) multilocus sequence analysis (MLSA) scheme. The *glnA, gyrB*, and *rpoD* genes of H2 were highly similar to those of P. putida LMG 14676. Previously, strain LMG 14676 was regarded as a member of P. putida biotype A based on phenotypic data (5) and SDS PAGE profiles of whole cell proteins (6), but it clearly separates from the P. putida type strain on the basis of ribopatterning (7). The *rpoB* gene sequence of strain H2 was incongruent to the *glnA, gyrB*, and *rpoD* gene sequences. Comparison among the 11 completely sequenced P. putida chromosomes showed that the H2 genome, estimated at 5.79 Mb, is with one exception, smaller than all P. putida genomes (the median for P. putida is 6.03 Mb, and the smallest and largest genomes are 5.73 and 6.87 Mb). Unsurprisingly, the H2 genome contains fewer predicted protein coding sequences (CDSs) (4,985) than most P. putida genomes (the median number of CDSs is 5,321, and the minimum and maximum are 4,960 and 6,357). Finally, the G+C contents of the H2 genome vary between 28 and 75%, with a median of 62.6% (500-bp sliding window). Combined with the MLSA results, these data suggest that the H2 genome has been considerably impacted by horizontal gene transfer. Because of the few clear distinctions from P. putida, we define strain H2 here as a Pseudomonas sp. nov.

### Nucleotide sequence accession numbers.

This whole-genome shotgun project has been deposited at DDBJ/EMBL/GenBank under the accession no. JRPO00000000. The version described here is version JRPO01000000. Strain H2 is available from the LMG culture collection (http://bccm.belspo.be/about/lmg.php, LMG 28719).
